# Functional characterization of RhuB as a second TonB2-dependent hemin receptor in *Riemerella anatipestifer* CH-1

**DOI:** 10.1128/spectrum.03133-23

**Published:** 2024-02-20

**Authors:** Mengying Wang, Siyi Wang, Mingshu Wang, Dekang Zhu, Renyong Jia, Shun Chen, Xinxin Zhao, Qiao Yang, Ying Wu, Shaqiu Zhang, Juan Huang, Mafeng Liu, Anchun Cheng

**Affiliations:** 1 Engineering Research Center of Southwest Animal Disease Prevention and Control Technology, Ministry of Education of the People’s Republic of China, Chengdu, China; 2 Key Laboratory of Animal Disease and Human Health of Sichuan Province, Chengdu, China; 3 International Joint Research Center for Animal Disease Prevention and Control of Sichuan Province, Chengdu, China; 4 Research Center of Avian Disease, College of Veterinary Medicine, Sichuan Agricultural University, Chengdu, China; Griffith University - Gold Coast Campus, Gold Coast, Australia

**Keywords:** *Riemerella anatipestifer*, TonB2-dependent receptor, RhuB

## Abstract

In the previous study, it was shown that *Riemerella anatipestifer* (*R. anatipestifer*, RA), a pathogen in ducks and some other birds, encodes a hemin uptake system. The *R. anatipestifer* hemin uptake receptor RhuR is a TonB2-dependent hemin transporter. However, it remains unclear whether *R. anatipestifer* encodes additional TonB-dependent hemin transporters. Herein, we demonstrated that *R. anatipestifer* hemin uptake receptor B (RhuB) of *R. anatipestifer* CH-1 (RA CH-1) was negatively regulated by iron and mediated by the Fur protein, and knocking out *rhuB* damaged the ability of RA CH-1 to utilize iron from duck hemoglobin (Hb) but not that from duck serum. Moreover, the ability to use iron from Hb was restored by the expression *rhuB in trans*. Furthermore, the RhuB of RA CH-1 is a membrane protein, and recombinant RhuB could bind hemin at a 1:1 molar ratio *in vitro*. Compared to that of Δ*tonB1*Δ*rhuR*, the ability of Δ*tonB1*Δ*rhuR*Δ*rhuB* to utilize hemin was impaired; meanwhile, compared to that of Δ*tonB2*Δ*rhuR*, the hemin utilization ability of Δ*tonB2*Δ*rhuR*Δ*rhuB* was not affected, indicating that RhuB is a TonB2-dependent receptor. Compared to Δ*rhuB*, *ΔrhuBΔrhuA* did not affect hemin utilization. However, compared to Δ*rhuA*, *ΔrhuBΔrhuA* had reduced ability to utilize hemin, suggesting that RhuA relies on RhuB for its activity. Finally, the deletion of *rhuB* did not affect the virulence of RA CH-1. These results suggested that RhuB encodes a TonB2-dependent hemin receptor. The characterization of the second TonB-dependent receptor in *R. anatipestifer* enriches our understanding of the hemin uptake system of this bacterium.

**IMPORTANCE:**

Iron is essential for the survival of most bacteria, and hemin of hemoglobin can serve as an important iron source. In our previous studies, we showed that *R. anatipestifer* CH-1 encodes a TonB2-dependent hemin receptor RhuR, which is involved in hemin uptake. The deletion of *rhuR* did not abolish hemin utilization by RA CH-1. We hypothesized that additional hemin uptake systems exist in this bacterium. In this study, we identified the second TonB2-dependent hemin receptor RhuB in RA CH-1 through hemin utilization, protein localization, and hemin-binding experiments. The duck infection model showed that the deletion of *rhuB* did not affect the virulence of RA CH-1. This study is not only important for further understanding the hemin utilization mechanism of *R. anatipestifer*, but also for enriching the hemin uptake transporters of gram-negative bacteria.

## INTRODUCTION

Iron is an essential metal for most organisms, including *Riemerella anatipestifer* (*R. anatipestifer*, RA) (1), because of its role as a cofactor for metabolic enzymes, oxidoreductases, electron transport chain components, and DNA biosynthesis (2). However, under aerobic conditions, iron is oxidized to Fe^3+^, forming insoluble hydroxide ion polymers (3). In vertebrate hosts, most iron is found in the form of hemin, which binds to hemoglobin (Hb), myoglobin, hemopexin, albumin, and cytochromes (4). Residual Fe^3+^ is chelated by transferrin in the serum and lactoferrin in mucus and tears, etc. (2).

To overcome this obstacle, some bacteria secrete siderophores to mobilize iron from their hydroxide polymers or host iron-binding proteins, such as transferrin (2). Fe-bound siderophores are transported across the bacterial outer membrane by specific TonB-dependent transporters, whereas ATP-binding cassette (ABC) transporters are responsible for the transportation of Fe-bound siderophores through the inner membrane. In the cytoplasm, iron is released from the siderophores by siderophore-interacting proteins (5). For hemin utilization, gram-negative bacteria encode specific TonB-dependent hemin receptor(s) for transport, which are degraded by hemin oxygenase or hemin-degrading proteins to release iron (6). To maintain iron homeostasis in cells, genes encoding iron or hemin uptake systems in most gram-negative bacteria are transcriptionally regulated by iron in a pathway mediated by the Fur protein (4, 7). When iron levels are too high in the cytoplasm, iron-bound Fur binds to the promoter region of iron or hemin transporter genes to repress gene transcription. Conversely, lower external iron can cause the dissociation of Fur from the promoter region of iron or hemin transporter genes to promote gene transcription (4). Because free iron and hemin are almost nonexistent in the host, these uptake systems are important for bacterial pathogens during infection.

*R. anatipestifer* is a gram-negative bacterial pathogen that mainly infects ducks, geese, and other birds and belongs to the family *Weeksellaceae* (8). Currently, more than 21 serotypes have been identified, and there is no cross-protection between them (9, 10). Although some genes, such as *B739_1208* (11), *B739_1343* (12), *wza*-like gene (13), type IX secretion system (T9SS) genes (14, 15), T9SS effector subsp. (16), endonuclease AS87_RS02955 (17), Fur (7, 18), PhoP (19), and OMP76 (20) have been identified as virulence factors of *R. anatipestifer,* the pathogenic mechanism of this bacterium remains largely unknown.

*R. anatipestifer* genome encodes at least 31 putative TonB-dependent receptors (TbdRs) (21); however, the physiological roles of most of these putative TbdRs remain largely unknown. In our previous study, we showed that the outer membrane hemin-binding protein RhuA participates in hemin transport through the TonB2-dependent hemin receptor RhuR in *R. anatipestifer* CH-1 (22). In addition to RhuR, other TonB-dependent receptors, such as *B739_1068* in RA CH-1, are upregulated under iron-limited conditions (23). In this study, we aimed to show that the *B739_1068* of RA CH-1 is a TonB2-dependent membrane protein, and B739_1068 could utilize hemin from duck Hb, which has been renamed as RhuB (*R. anatipestifer* hemin uptake receptor B).

## RESULTS AND DISCUSSION

### Sequence analysis of *rhuB*

In a previous study, RNA-seq data showed that the B739_1068 transcript is upregulated under iron-limited conditions (23). The promoter region of *B739_1068* contains a putative Fur box (7). However, sequence comparison showed that B739_1068 had low identity (<20%) with well-characterized FecA proteins, such as FecA of *Escherichia coli*, FecA of *Pseudomonas aeruginosa,* and FecA of *Hafnia paralvei* (data not shown). CLUSTALW analysis comparing B739_1068 with well-characterized TonB-dependent hemin receptors, including HemR of *Serratia marcescens*, HasR of *Serratia marcescens*, HpuB of *Neisseria meningitidis*, HmuR of *Porphyromonas gingivalis*, RhuR of *R. anatipestifer,* HuxC of *Haemophilus influenzae* and ChuA of *Escherichia coli*, showed identity rates in the 12%–17% range. Thus, it was impossible to determine whether B739_1068 functions as an iron compound receptor or as a hemin receptor through sequence analysis (data not shown). Based on these data, as well as the data described below, B739_1068 is designated as RhuB (*R. anatipestifer* hemin uptake receptor B).

### *R. anatipestifer* CH-1 gene *rhuB* is regulated by iron and Fur

**Fig 1:**
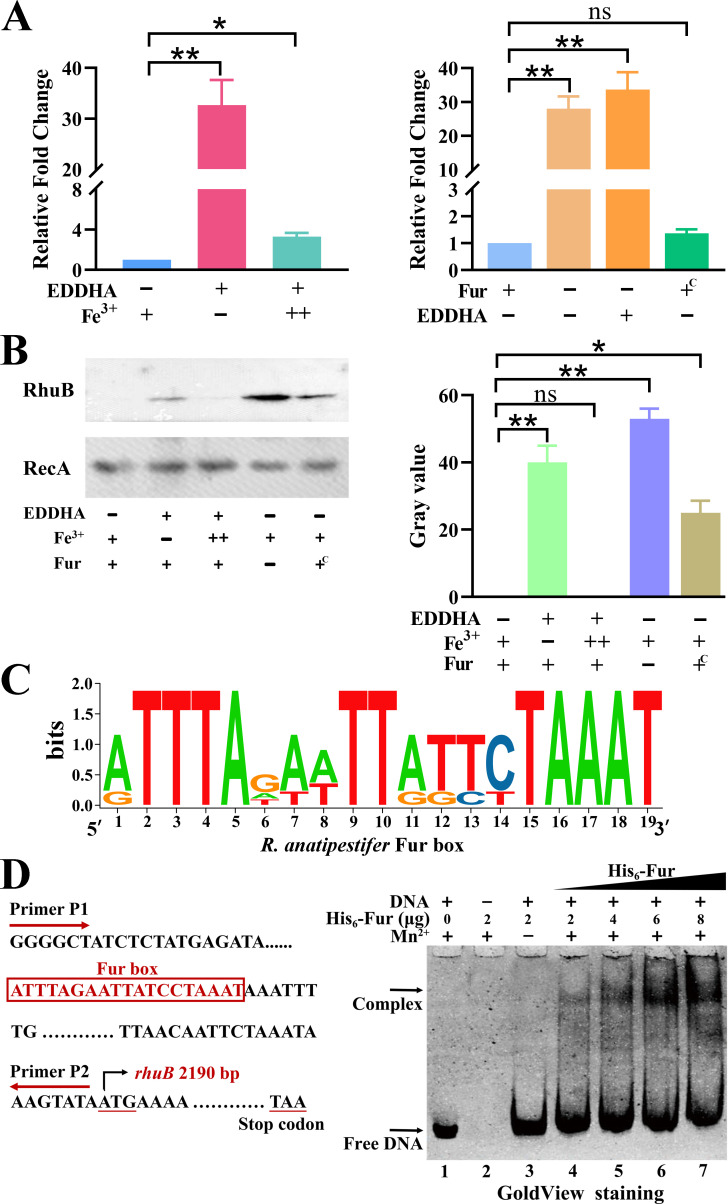
Expression rate of *rhuB* is regulated by iron and Fur levels. (**A**) Left, qRT-PCR analysis of the fold change in the iron-responsive transcript levels of the *rhuB* gene in RA CH-1 grown in the GCB medium (column 1), GCB medium containing 120 µM EDDHA (column 2) or 120 µM EDDHA together with 100 µM Fe(NO_3_)_3_ (column 3). Right, The transcript levels of *rhuB* in RA CH-1 pLMF03 grown in GCB medium (column 1) and in RA CH-1Δ*fur* pLMF03 grown in the GCB (column 2) and GCB medium supplemented with 120 µM EDDHA (column 3), and the transcript levels of *rhuB* in RA CH-1Δ*fur* pLMF03::*fur* in the GCB medium (column 4). The values shown are the averages and standard deviations derived from three experiments. Asterisks denote significant differences (*P < 0.05, **P < 0.01) between two groups; n.s, not significant. (**B**) Left, western blot detection of RhuB in lysates from RA CH-1 pLMF03 grown in the GCB medium (Lane 1), in GCB medium combined with 120 µM EDDHA (Lane 2), in GCB medium together with 120 µM EDDHA supplemented with 100 µM Fe(NO_3_)_3_ (Lane 3); lysates from RA CH-1Δ*fur* pLMF03 (Lane 4) and RA CH-1Δ*fur* pLMF03::*fur* grown in the GCB medium (Lane 5). All samples were subjected to SDS-PAGE and RhuB was detected using a specific anti-RhuB antibody. RecA was used as the internal reference. Right: RhuB protein expression in RA CH-1 derivative strains grown under different conditions. The intensity of the RhuB band was measured using ImageJ software. (**C**) Sequence logo of a Fur box in *R. anatipestifer*. (**D**) Left, identification of a Fur box at the *rhuB* promoter region, indicated in red. Right, binding of Fur to the *rhuB* promoter, as examined by EMSA, escalating quantities of purified His_6_-Fur protein.

Previous transcriptome findings have shown that *B739_1068* (*rhuB*) in RA CH-1 is an iron-regulated gene (23). Herein, we aimed to examine whether it was also regulated by Fur. Iron-limited conditions significantly upregulated the transcription rate of *rhuB* compared to iron-rich conditions, and this induction was repressed by exogenous Fe(NO_3_)_3_ (Fig. 1A). The transcript level of the *rhuB* gene increased more than 20-fold in the *fur* mutant strain compared to that in the wild-type (WT) strain, and the addition of EDDHA did not affect the transcription rate of *rhuB* in the *fur* mutant strain (Fig. 1A). Moreover, the increased transcript levels were fully restored to the WT levels by complementation with *fur* (Fig. 1A). The negative regulatory effect of iron and Fur on *rhuB* was further confirmed at the protein level with the observation that RhuB production was significantly elevated in the iron-limited condition and *fur* mutant strain compared with that in the WT and complemented strains, respectively (Fig. 1B; Fig. S1). Sequence comparison shows that the promoter region of *rhuB* contains an *R. anatipestifer* Fur box (5′-ATTTAGAATTATCCTAAAT-3′) (7). The motif was visualized using the TBtools software (24) (Fig. 1C). To further demonstrate that the Fur protein has a direct interaction with the promoter region of *rhuB* (194 bp), an electrophoretic mobility shift assay (EMSA) experiment was performed as described in a previous study (7). The presence of Fe^2+^ was necessary for the DNA-binding activity of Fur, but Fe^2+^ is easily oxidized to Fe^3+^ when exposed to air. Thus, Mn^2+^ was used to substitute for Fe^2+^ (7, 25). As shown in Fig. 1D, the formation of DNA–Fur protein complexes was observed only when Mn^2+^ was present and the binding was dependent on the concentration of Fur protein. Using specific binding with the Hill slope equation (Graphpad prism V.9) to determine a binding affinity constant (*K*_d_), we derived the value of 4.3 × 10^−9^ M for the recombinant Fur-binding promoter region of *rhuB* (26). These results suggested that *rhuB* expression is directly regulated by Fur.

### RhuB was involved in hemin utilization from duck Hb

**Fig 2:**
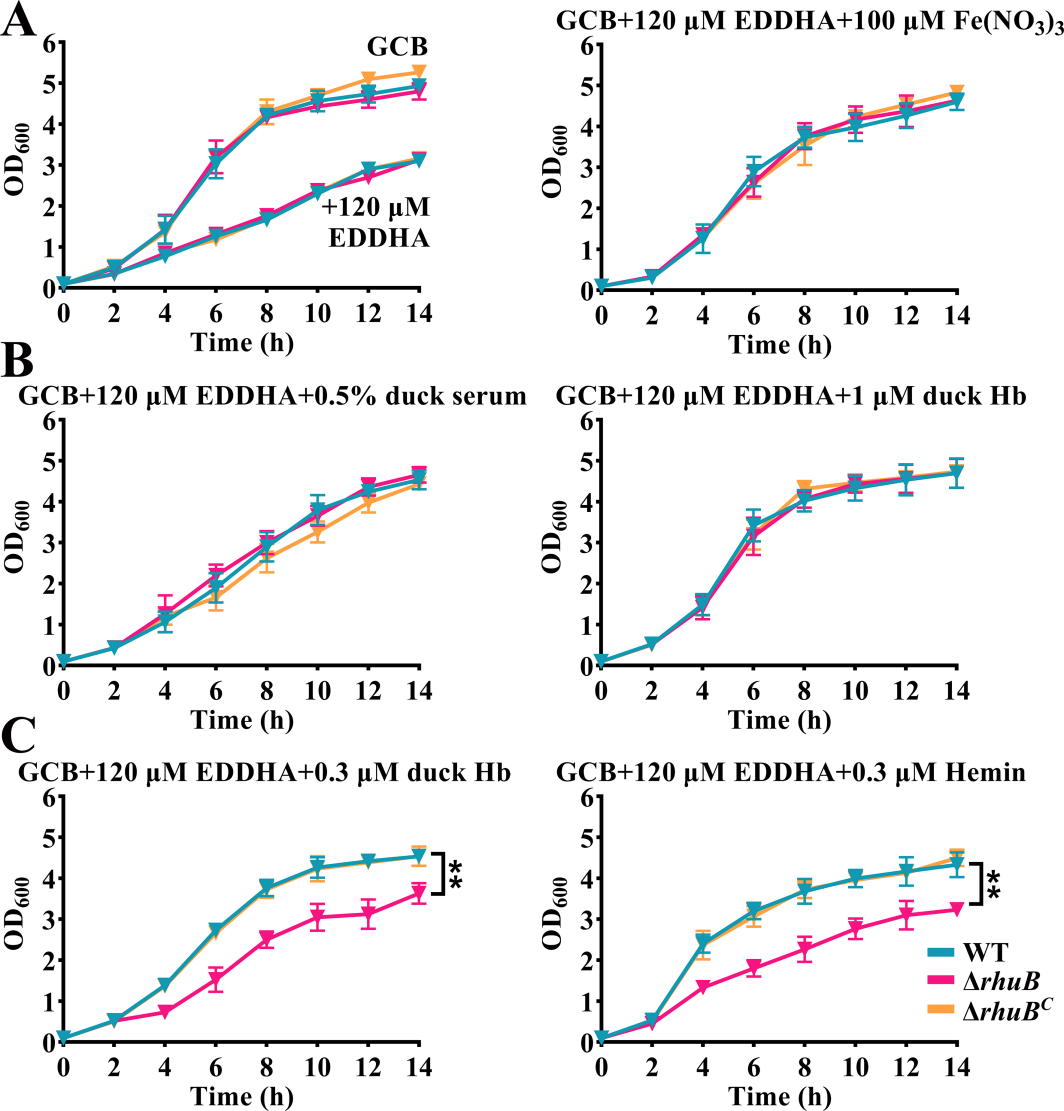
Growth curves of *R. anatipestifer* strains under different iron source conditions. (**A**) The growth curve of RA CH-1 pLMF03 (WT), RA CH-1Δ*rhuB* pLMF03 (Δ*rhuB*) and RA CH-1Δ*rhuB* pLMF03::*rhuB* (Δ*rhuB^C^*) in the GCB liquid medium and GCB combined with 120 µM EDDHA (left), GCB medium combined with 120 µM EDDHA supplemented with 100 µM Fe(NO_3_)_3_ (right). (**B**) The growth curve of these strains in the GCB medium together with 120 µM EDDHA supplemented with 0.5% inactivated duck serum (left) or 1 µM duck hemoglobin (Hb) (right). (**C**) The growth curve of these strains in the GCB medium together with 120 µM EDDHA supplemented with 0.3 µM duck hemoglobin (left) or 0.3 µM hemin (right). The values shown are the averages and standard deviations derived from three experiments.

Because RhuB, which encodes a putative TonB-dependent receptor, was regulated by iron and Fur, it was proposed that RhuB was involved in iron or hemin transportation. Thus, we identified the growth effect of RA CH-1Δ*rhuB* (Δ*rhuB*) in the iron-limited medium supplemented by Fe(NO_3_)_3_, where inactivated duck serum or duck Hb served as the sole iron source, respectively. As shown in Fig. 2A, the mutant of *rhuB* did not affect the growth of RA CH-1 (WT) in a GCB medium or GCB medium containing the iron chelator EDDHA. Using 100 µM Fe(NO_3_)_3_ or 0.5% inactivated duck serum as a sole iron source, the growth of all bacteria was restored and no difference in growth rates was observed (Fig. 2A and B). Similarly, a lower concentration of Fe(NO_3_)_3_ or inactivated duck serum as the sole iron source did not lead to significant growth differences among the bacterial strains (data not shown). Meanwhile, the addition of 1 µM duck Hb restored the growth of all strains in an iron-limited medium (Fig. 2B). However, duck Hb supplementation at 0.3 µM or hemin supplementation at 0.3 µM restored the growth of the WT and complemented strains but did not restore the growth of the *rhuB* mutant strain in iron-limited medium (Fig. 2C). These results suggest that *rhuB* is required for hemin utilization from duck Hb but is not involved in iron utilization from duck serum.

### RhuB is a membranous hemin-binding protein

**Fig 3:**
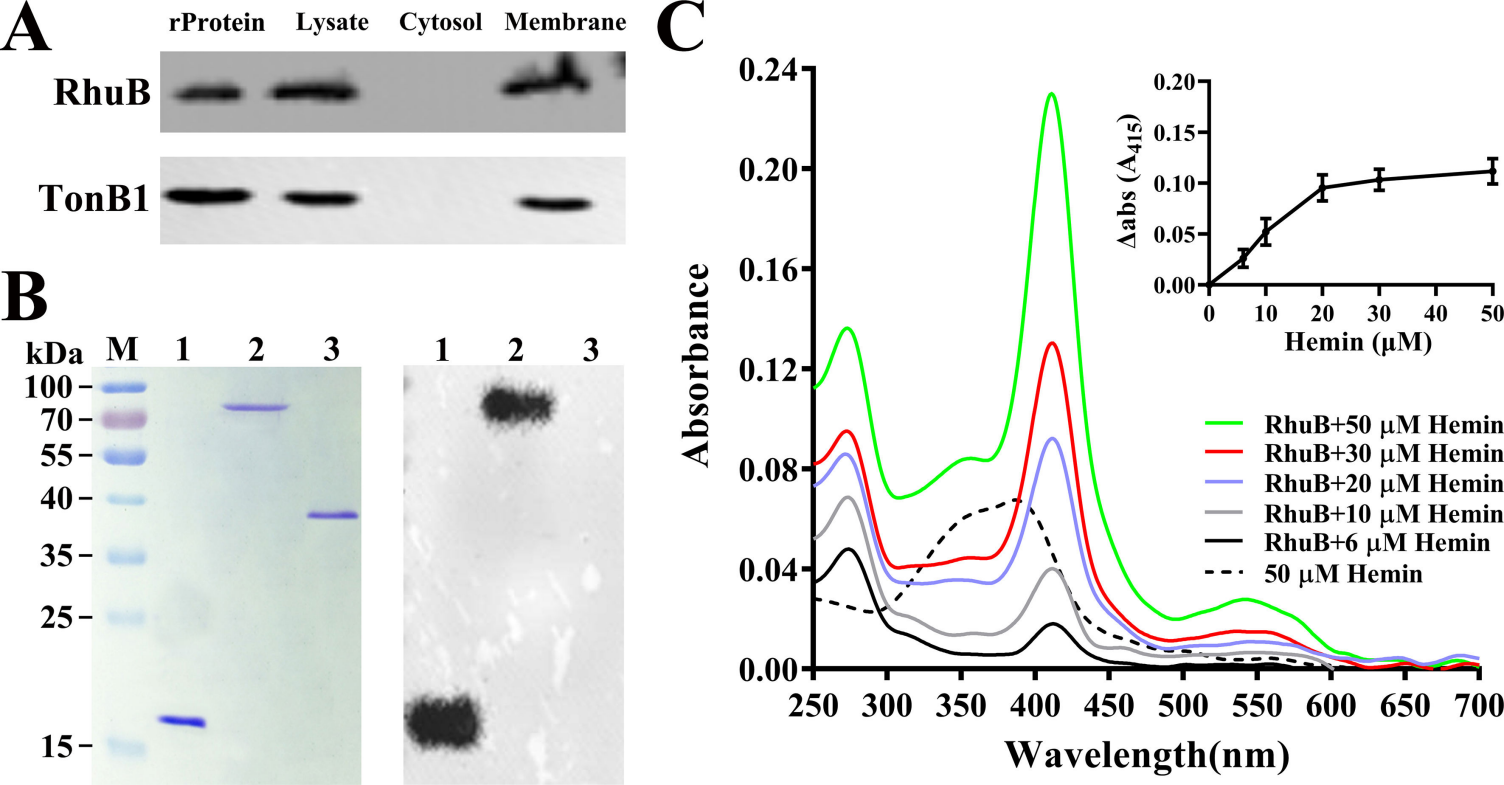
Subcellular location of RhuB in *R. anatipestifer* CH-1 and the hemin-binding assay of the recombinant RhuB. (**A**) RhuB was detected in the RA CH-1 lysate, cytosol, and membrane using a polyclonal antibody. The subcellular localization of the membrane protein TonB1 in RA CH-1 cells was used as a positive control. rProtein indicates the purified recombinant protein used for comparison. (**B**) Left, recombinant HasA of *Serratia marcescens* (Lane 1; positive control), recombinant RhuB (Lane 2), and recombinant TonB1 (Lane 3; negative control) were run on a polyacrylamide gel. The gels were stained with Coomassie brilliant blue. Right, the proteins were transferred to a nitrocellulose filter and subjected to hemin blotting. The experiment was repeated three times, and independent experiments were performed. (**C**) Increasing concentrations of hemin were added to 20 µM recombinant RhuB, and the absorption spectrum from 250 to 700 nm was measured using nanodrop 2000. The spectrum corresponding to 50 µM hemin was used as a control. The inset shows the absorbance values of hemin–rRhuB minus those of hemin alone at 415 nm with increasing hemin concentrations.

Because RhuB is involved in the utilization of hemin from duck Hb, it may be a hemin-binding protein. First, the subcellular distribution of this protein in RA CH-1 was determined. Membrane and cytosolic fractions of RA CH-1 were prepared, and RhuB was detected by western blotting using a specific antibody against RhuB. RhuB was detected exclusively in membrane fractions (Fig. 3A; Fig. S2). To identify the potential physical interactions between recombinant RhuB and hemin, the recombinant proteins were separated by SDS-PAGE and transferred to a nitrocellulose filter for hemin blotting. This method has been used to detect the binding of hemin to cytochrome C (27) and the ISD system of *Staphylococcus aureus* (28). Similar to recombinant HasA of *Serratia marcescens* (positive control), recombinant RhuB was able to bind to hemin (Fig. 3B). In contrast, the recombinant TonB1 protein from RA CH-1 (negative control) was unable to bind to hemin under these conditions (Fig. 3B). When the protein binds to hemin, it produces a specific Soret band (29). Therefore, hemin binding to rRhuB was assessed spectrophotometrically. As shown in Fig. 3C, recombinant RhuB (20 µM) was incubated with different concentrations of hemin (6, 10, 20, 30, and 50 µM), resulting in the appearance of a Soret peak at 415 nm, indicating that RhuB–hemin complex formation. The inset in Fig. 3C shows the absorbance values of hemin–rRhuB minus those of hemin alone at 415 nm with increasing hemin concentrations. Plotting the spectral changes observed at 415 nm versus hemin concentration yielded a saturation curve (Fig. 3C). When the hemin concentration is less than 20 µM, the absorbance at 415 nm increases with the increase of the hemin concentration, while when the hemin concentration is greater than 20 µM, the absorbance at 415 nm does not increase (Fig. 3C), indicating that the binding of hemin to the recombinant RhuB depends on the concentration and may occur at a 1:1 molar ratio. Collectively, these findings suggested that *rhuB* of RA CH-1 encodes a membranous hemin-binding protein.

### Function of RhuB is TonB2 dependent

*R. anatipestifer* encodes three TonB proteins: TonB1, TonB2, and TonB3 (TbfA) (1, 30). Both TonB1 and TonB2 are involved in hemin utilization, but not TonB3 (TbfA) (1, 30). Herein, we investigated whether RhuB functions in a TonB1 or TonB2 manner. Thus, we constructed the strains RA CH-1Δ*tonB1*Δ*rhuB* pLMF03 (Δ*tonB1*Δ*rhuB*), RA CH-1Δ*tonB1*Δ*rhuB* pLMF03::*rhuB* (Δ*tonB1*Δ*rhuB^C^*), RA CH-1Δ*tonB2*Δ*rhuB* pLMF03 (Δ*tonB2*Δ*rhuB*), and RA CH-1Δ*tonB2*Δ*rhuB* pLMF03::*rhuB* (Δ*tonB2*Δ*rhuB^C^*) and evaluated their hemin utilization capacity. The results revealed that the knockout of *rhuB* did not affect the hemin utilization of Δ*tonB1* and Δ*tonB2* (Fig. S3).

**Fig 4:**
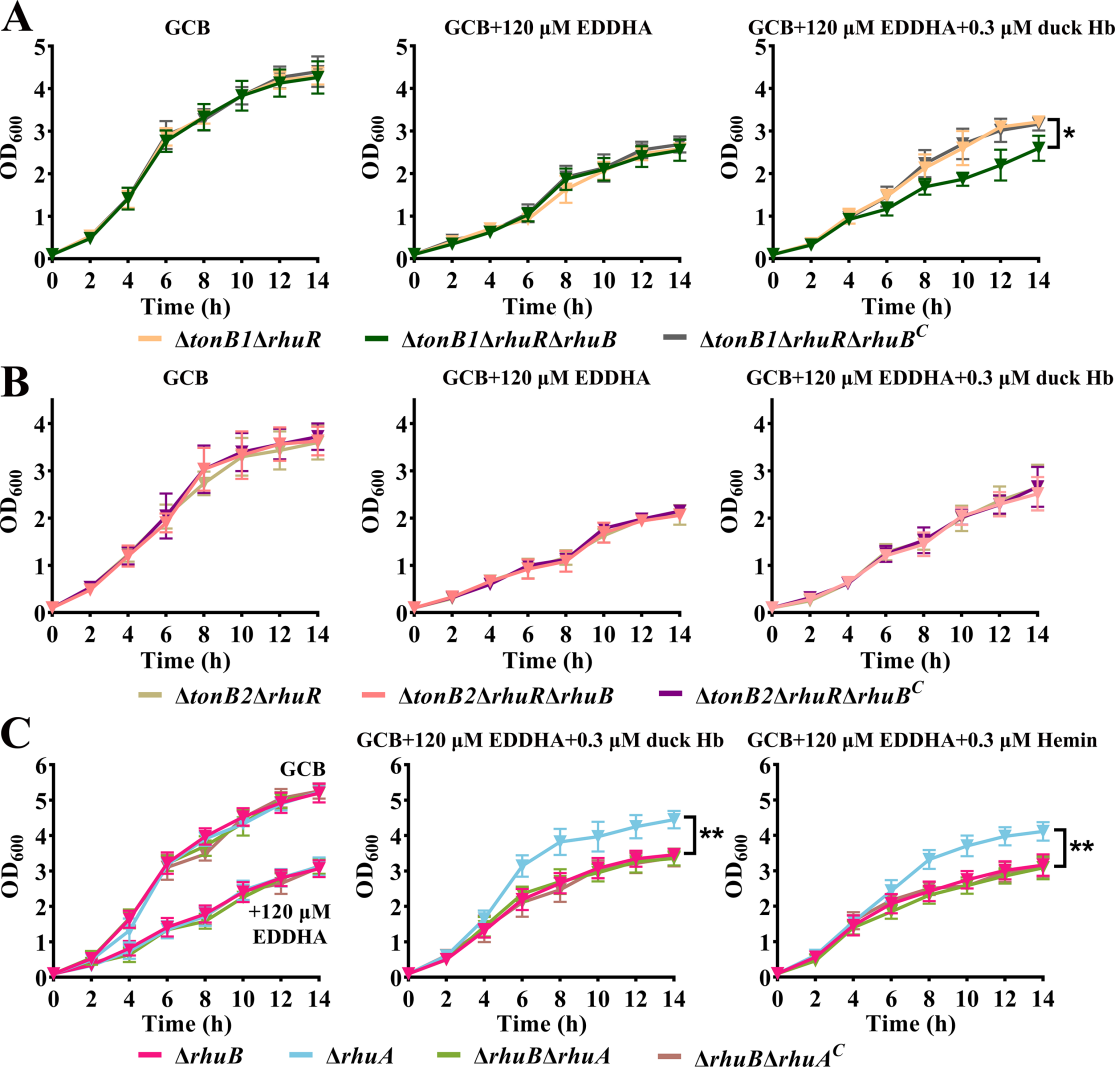
*rhuB* depends on TonB2 to participate in the utilization of duck hemoglobin. (**A**) The growth curves of RA CH-1Δ*tonB1*Δ*rhuR* pLMF03 (Δ*tonB1*Δ*rhuR*), RA CH-1Δ*tonB1*Δ*rhuR*Δ*rhuB* pLMF03 (Δ*tonB1*Δ*rhuR*Δ*rhuB*), and RA CH-1Δ*tonB1*Δ*rhuR*Δ*rhuB* pLMF03::*rhuB* (Δ*tonB1*Δ*rhuR*Δ*rhuB^C^*) in the GCB liquid medium, GCB combined with 120 µM EDDHA, and GCB combined with 120 µM EDDHA and supplemented with 0.3 µM duck hemoglobin. (**B**) The growth of RA CH-1Δ*tonB2*Δ*rhuR* pLMF03 (Δ*tonB2*Δ*rhuR*), RA CH-1Δ*tonB2*Δ*rhuR*Δ*rhuB* pLMF03 (Δ*tonB2*Δ*rhuR*Δ*rhuB*), and RA CH-1Δ*tonB2*Δ*rhuR*Δ*rhuB* pLMF03::*rhuB* (Δ*tonB2*Δ*rhuR*Δ*rhuB^C^*) in the GCB liquid medium, GCB combined with 120 µM EDDHA, and GCB combined with 120 µM EDDHA supplemented with 0.3 µM duck hemoglobin. (**C**) The growth curves of the Δ*rhuB*, RA CH-1Δ*rhuA* pLMF03 (Δ*rhuA*), RA CH-1Δ*rhuB*Δ*rhuA* pLMF03 (Δ*rhuB*Δ*rhuA*), and RA CH-1Δ*rhuB*Δ*rhuA* pLMF03::*rhuA* (Δ*rhuB*Δ*rhuA^C^*) in the GCB liquid medium, GCB combined with 120 µM EDDHA, GCB combined with 120 µM EDDHA and supplemented with 0.3 µM duck hemoglobin or 0.3 µM hemin. The values shown are the averages and standard deviations derived from three experiments.

Furthermore, we deleted *rhuB* in strains RA CH-1Δ*tonB1*Δ*rhuR* pLMF03 (Δ*tonB1*Δ*rhuR*) and RA CH-1Δ*tonB2*Δ*rhuR* pLMF03 (Δ*tonB2*Δ*rhuR*), and evaluated their hemin utilization capacity. As shown in Fig. 4A, compared to Δ*tonB1*Δ*rhuR*, RA CH-1Δ*tonB1*Δ*rhuR*Δ*rhuB* pLMF03 (Δ*tonB1*Δ*rhuR*Δ*rhuB*) exhibited a significant growth delay in GCB supplemented with 120 µM EDDHA and 0.3 µM duck Hb. However, the deletion of *rhuB* did not affect hemin utilization of Δ*tonB2*Δ*rhuR* (Fig. 4B). This result suggests that RhuB functions as a TonB2-dependent hemin receptor. It further indicates that RhuB is the second TonB2-dependent hemin receptor.

In our previous study, we showed that the function of the outer membrane hemin-binding protein, RhuA, is dependent on RhuR (22). We further investigated whether RhuA is dependent on RhuB. Therefore, we constructed the strain RA CH-1Δ*rhuB*Δ*rhuA* (Δ*rhuB*Δ*rhuA*) and evaluated its hemin utilization capacity. As shown in Fig. 4C, when duck Hb or hemin was the sole iron source, the Δ*rhuB*Δ*rhuA* exhibited a significant growth deficiency compared to the Δ*rhuA*. Conversely, there was no noticeable difference in growth rates between the Δ*rhuB*Δ*rhuA* and Δ*rhuB* under the same condition (Fig. 4C). These results suggested that RhuA transfers hemin to RhuB.

### RhuB mutant has no effect on *R. anatipestifer* virulence

**Fig 5:**
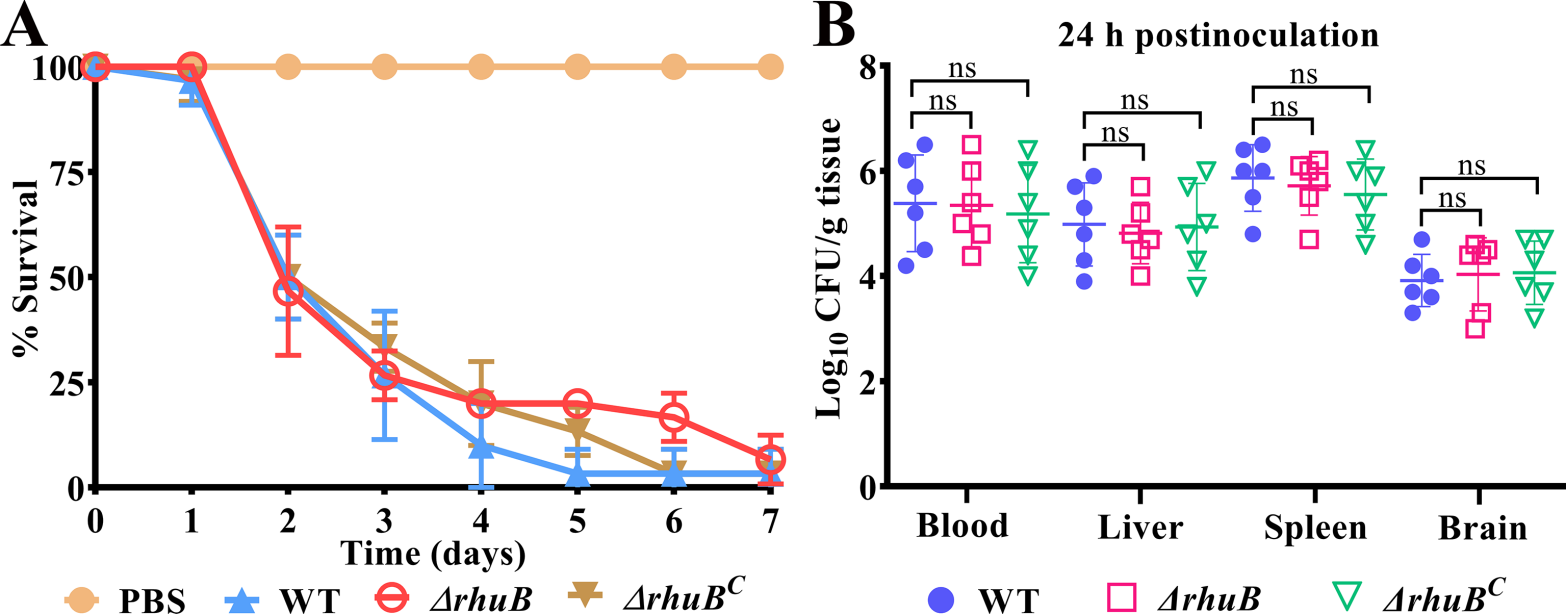
Virulence and colonization evaluation of RA CH-1 and RA CH-1*∆rhuB*. (**A**) Survival rate of ducklings infected with 10^9^ CFU of RA CH-1 pLMF03 (WT), RA CH-1Δ*rhuB* pLMF03 (Δ*rhuB*), RA CH-1Δ*RhuB*pLMF03::*rhuB* (Δ*rhuB^C^*). PBS was used as a negative control. (**B**) Three-day-old ducklings were infected with 5 × 10^8^ CFU of WT, Δ*rhuB*, Δ*rhuB^C^*. After 24 h, the bacterial loads in different tissues were recorded. Differences were assessed for statistical significance using the Student’s *t*-test.

Hemin uptake systems are required for the virulence of many bacterial pathogens (31). To investigate the role of the RhuB-dependent hemin transport system in the pathogenesis, RA CH-1 pLMF03 (WT), RA CH-1Δ*rhuB* pLMF03 (Δ*rhuB*), and RA CH-1Δ*rhuB* pLMF03::*rhuB* (Δ*rhuB*^C^) were prepared to infect 3-day-old ducklings. As shown in Fig. 5, the survival rate of Δ*rhuB* was not significantly different from that of the parental strain. Moreover, at 24 h postinfection, compared with the WT, Δ*rhuB* had colonized the ducklings at similar levels (Fig. 5). Furthermore, we compared the pathogenicity of the WT and *ΔrhuBΔrhuR* double mutants in the ducklings. The results showed no significant differences in mortality rates or colonization levels between the two strains (data not shown). These data suggested that the *rhuB* mutant had no effect on *R. anatipestifer* virulence.

### Distribution of the RhuB homologues

**Fig 6:**
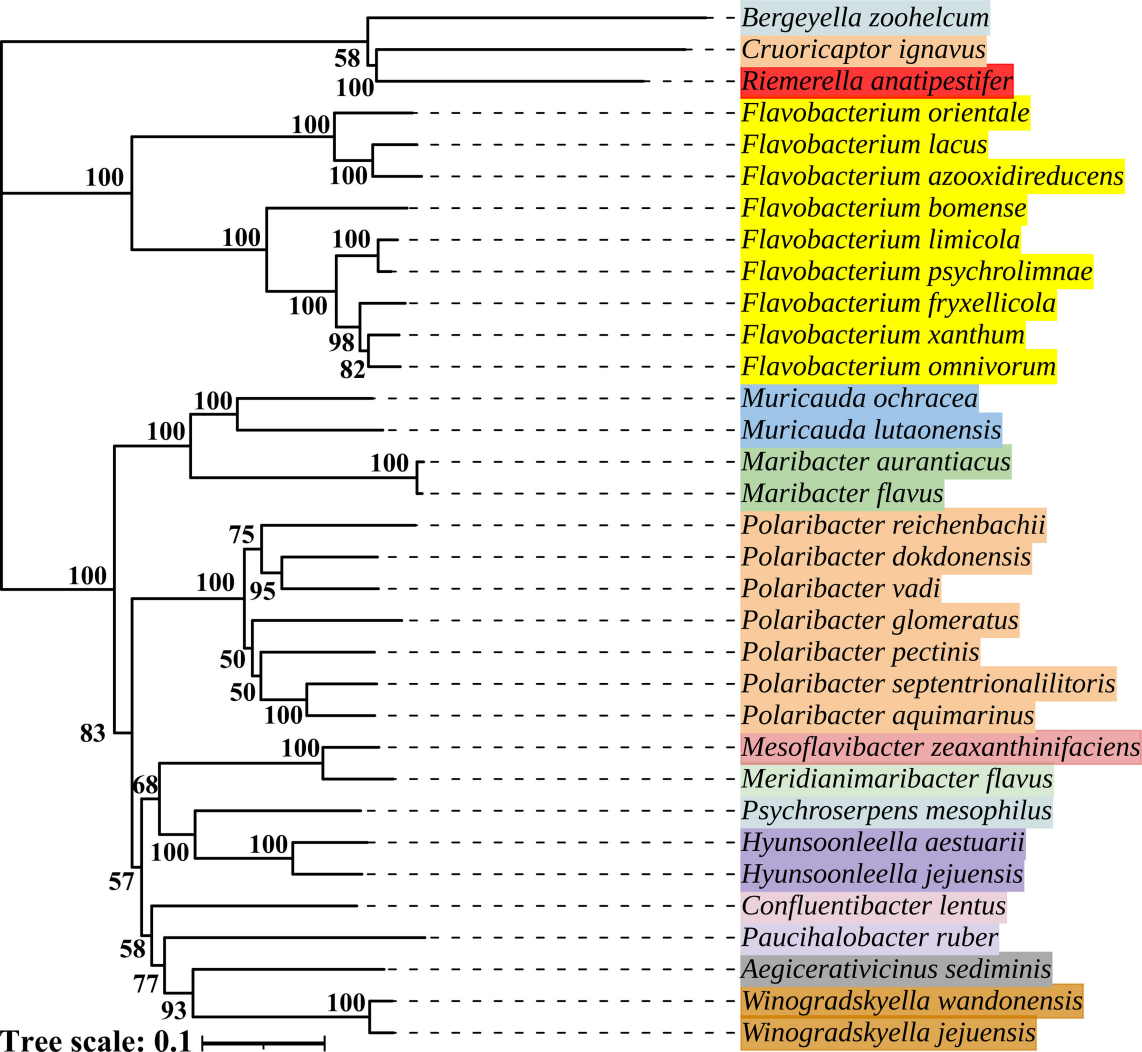
Systematic evolutionary tree of RhuB and its homologous proteins. The systematic evolution analysis of RhuB is highlighted in red. A systematic evolutionary tree was constructed using the neighbor-joining method in the MEGA7 software. The scale bar represents the percentage divergence (distance). The numbers represent bootstrap values.

Sequence comparison showed that the RhuB protein exhibited low sequence identity with the characterized TonB-dependent hemin receptors of *R. anatipestifer*, including the RhuR of RA CH-1 (24.63% similarity and 15.8% identity) and TbdR1 of *R. anatipestifer* CH3 (28% similarity and 15.17% identity). This evidence represented a newly identified TonB2-dependent hemin receptor in *R. anatipestifer*. Sequence analysis showed that RhuB was highly conserved in all sequenced *R. anatipestifer* strains, with 95.61%–99.86% identity. In addition, RhuB exhibits 42%–62.16% amino acid similarity to other uncharacterized proteins annotated as putative TonB-dependent receptors (TBDRs) in *Flavobacterium lacus, Muricauda ochracea*, *Polaribacter vadi*, *Maribacter flavus*, and *Bergeyella zoohelcum* (Fig. 6; Fig. S4).

### Conclusion

In this study, we identified a novel hemin TonB-dependent receptor RhuB, whose expression is regulated by iron and Fur protein levels. Moreover, this study showed that RhuB function is contingent on the presence of TonB2. Despite its significant role in hemin utilization, *rhuB* mutation did not notably affect the virulence of *R. anatipestifer*, indicating the presence of other compensatory hemin acquisition mechanisms that sustain its pathogenicity. These insights into the role and regulation of RhuB in *R. anatipestifer* enhance our understanding of bacterial hemin utilization.

## MATERIALS AND METHODS

### Bacterial strains, plasmids, and primers

The bacterial strains and plasmids used in this study are listed in Table S1. Primers used are listed in Table S2.

### Growth conditions

Iron chelator, ethylenediamine-*N*,*N*′-bis [(2-hydroxyphenyl) acetic acid] (EDDHA), was purchased from Alfa Chemistry, Protheragen Inc. (USA). *Escherichia coli* strains were aerobically grown in an LB medium at 37°C. *R. anatipestifer* strains were cultured on LB agar plates supplemented with 5% sheep blood or in a Gonorrhoeae-Culture broth (GCB) liquid medium aerobically at 37°C (32). The solid medium contained 1.5% Difco agar. Antibiotics were used at the following concentrations when necessary: ampicillin (Amp) at 100 µg/mL and kanamycin (Kan) at 50 µg/mL for *E. coli*; cefoxitin (Cfx) at 1 µg/mL for *R. anatipestifer* CH-1.

### *In vitro* growth rate determination

The *in vitro* growth rates of the tested strains were determined by measuring the OD_600_ (optical density at 600 nm) using a spectrophotometer (Eppendorf Biophotometer, Germany), as described previously (30). Briefly, cultures in the early exponential phase were inoculated in 20 mL GCB liquid medium at an OD_600_ of 0.1 and incubated at 37°C with shaking (180 rpm). OD_600_ was determined every 2 h for 14 h.

### Preparation of cytoplasmic proteins and membrane proteins

Membrane fractions of RA CH-1 were prepared as previously described (22). Briefly, strain RA CH-1 was grown in 200 mL GCB liquid medium to an OD_600_ of 1. The cells were harvested by centrifugation at 8,000×*g* for 10 min and washed twice with PBS. The pellet was resuspended in 30 mL 20 mM Tris-HCl (pH 7.4)–10 mM EDTA-1 mM Na-p-tosyl-l-lysine chloromethyl ketone (TLCK) and lysed using a French press (Thermo). Cell debris was removed from the lysate by centrifugation at 8,000*×g* for 10 min at 4°C, and the supernatant was centrifuged at 100,000 g for 2 h at 4°C; 30 mL of the supernatant (cytosol) was transferred to a new tube. The pellet was resuspended in an equivalent volume of 1% *N*-lauryl sarcosine Na (Lot# SLBK2574 V; Sigma-Aldrich) and incubated for 1 h at room temperature as the membrane protein.

### Construction of the markerless mutant in *R. anatipestifer* CH-1

A series of genes (*rhuB* and *rhuA*) were deleted from RA CH-1 or *R. anatipestifer* derivative strains using a markerless deletion method, as described in a previous study (33). Primers used are listed in Table S2. Briefly, the upstream and downstream regions of the deleted region were amplified from the genome of RA CH-1 using the primers RhuB upP1 and RhuB upP2, RhuB downP1 and RhuB downP2, RhuA upP1 and RhuA upP2, RhuA downP1, and RhuA downP2 (Table S2). The two PCR fragments were ligated by overlapping PCR. The fused PCR fragment was cloned into the suicide vector, pOES (33). The recombinant plasmids were introduced into the CaCl_2_-competent strain *E. coli* S17-1 (34) and then transferred into strain RA CH-1 by conjugation, according to a previously described method (30). The transconjugants were selected from the GCB agar plates supplemented with Cfx (1 µg/mL) and Kan (50 µg/mL). The second homologous recombination was screened using 13 mM *p-Cl-Phe* counter selection, and the correct clone was identified as previously described (33).

### Construction of complementation plasmids and strains

Complementation plasmids were constructed based on the shuttle vector pLMF03 as described previously (30). Primers used are listed in Table S2. The plasmid pLMF03 derivatives were introduced into CaCl_2_-competent *E. coli* strain S17-1, and then transferred to *R. anatipestifer* strains by conjugation and selected on the blood plate containing 1 µg/mL Cfx, as described previously for complementation studies (30).

### Construction of the plasmids for protein expression

To express His-tagged recombinant RhuB, the complete RA CH-1 *rhuB* gene was amplified by PCR from RA CH-1 chromosomal DNA using the primers RhuB ExpP1 (introducing an *Nde*I site) and RhuB ExpP2 (introducing a *Kpn*I site and His tag) (Table S2). PCR fragments were purified, digested with the corresponding restriction endonucleases, and ligated into the plasmid pET32a, which had been digested with the same restriction endonucleases. Ligation mixtures were introduced into CaCl_2_-competent *E. coli* DH5α, and transformants were selected on LB plates containing Amp at 100 µg/mL. The colonies were screened by PCR using the corresponding primers. The validity of the sequence was determined using sequencing. The recombinant plasmids were transformed into *E. coli* BL21(DE3).

### Protein expression, purification, and antibody production

Protein expression strains JP313 pBAD24::*tonB1* (1), BL21(DE3) pET32a::*rhuB*, and BL21(DE3) pET32a::*fur* (7) were grown in 500 mL LB medium at 37°C to mid-log phase. Then, 0.02% arabinose (for JP313 pBAD24*::tonB1*) or 0.5 mM IPTG (for BL21 pET32a::*rhuB* and BL21 pET32a::*fur*) was added, and the cultures were further incubated for 3 h at 37°C before being harvested. The cultures were washed in 20 mL of binding buffer (50 mM Tris-HCl, 0.05% Triton, 250 mM NaCl, pH8.0) by centrifugation at 8,000×*g* for 10 min. The pellet was either stored at −20°C until use or resuspended in lysis buffer (binding buffer supplemented with 1 mg/mL lysozyme and 1 U/mL DNase I) for lysis. Cell debris was removed by centrifugation at 12,000×*g* at 4°C, and recombinant proteins were isolated as described in a previous study (1). Protein concentrations were determined using the Bradford assay according to the manufacturer’s instructions (Bio-Rad), with Bovine Serum Albumin as the standard. The purified His-tagged recombinant protein was used to produce polyclonal antibodies in mice using standard methods described in a previous study (1).

### Real-time RT-PCR

Real-time PCR was performed to evaluate the expression of *rhuB* in RA CH-1 cells. RNA was isolated using the RNeasy Mini Kit (Qiagen), and the isolated RNA was treated with DNase I (Qiagen) according to the manufacturer’s instructions. Real-time qPCR was performed as previously described (30). Fold change was calculated as described previously (35) using the delta-delta Ct method to consider the efficiency of the PCR reaction for each target. Quantitative measurements were performed on biological samples in triplicate, and the results were normalized to the RA CH-1 housekeeping gene 16S rDNA.

### Western blot analysis

Western blotting was performed as previously described (36) with slight modifications. Briefly, proteins were resolved by SDS-PAGE and transferred onto Polyvinylidene fluoride (PVDF) membranes (Millipore, Billerica, MA, USA). The membrane was blocked in 5% (w/v) nonfat milk powder overnight at 4°C and incubated with primary antibodies for 2 h at 37°C. The membrane was washed three times in TBST buffer (50 mM Tris, 150 mM NaCl, and 0.05% Tween 20, pH 7.4) and incubated with a 1:2,000 dilution of a goat anti-mouse IgG-coupled alkaline phosphatase-conjugated secondary antibody for 1 h at 37°C. Signals were detected using a BCIP/NBT solution, following the manufacturer’s instructions (Sigma), or enhanced chemiluminescence reagents (GE Healthcare ECL Plus) in the ChemiDoc MP Imaging System (Bio-Rad). The RecA antibody was prepared as previously described (30).

### Hemin-binding assay

The hemin-binding ability of RhuB was investigated using a previously described protocol with slight modifications (28). Briefly, proteins (1 µg) were mixed with 1 × loading buffer (200 mM Tris-HCl, 25% glycerin, 5% SDS, and 0.1% bromophenol blue, pH6.8) and separated using 12% SDS-PAGE. One gel was stained with Coomassie brilliant blue R250. Another gel was transferred to nitrocellulose, and the nitrocellulose membrane was washed for 10 min with TBST (10 mM Tris-HCl pH = 8.0, 150 mM NaCl, and 0.1% Tween 20) and subsequently probed for 1.5 h with TBS containing hemin (10^−6^ M) at room temperature. The nitrocellulose membrane was washed thrice for 10 min with TBST at room temperature. Hemin was visualized for its intrinsic peroxidase activity using enhanced chemiluminescence reagents (GE Healthcare ECL Plus) on a ChemiDoc MP Imaging System (Bio-Rad).

### Electrophoretic mobility shift assay

EMSA was performed as previously described, with minor modifications (7). Briefly, the promoter of *rhuB* (194 bp) was amplified from RA CH-1 genomic DNA using the primers RhuBpromoterP1 and RhuBpromoterP2. Each 20 µL EMSA reaction solution contained 1× binding buffer (2.5% glycerol, 0.05% NP-40, 5 mM MgCl_2_, 1 µM ZnSO_4_), 5 mM MnCl_2_ (37), a typically surrogate for iron to maintain the regulatory activity (7, 38). We used 1 µg DNA and increasing amount of purified His_6_-Fur protein (2, 4, 6, and 8 µg). After reacting for 20 min at room temperature, the protein–DNA mixture was separated on a 6% (w/v) polyacrylamide native gel and visualized by staining with 2% GoldView and Coomassie brilliant blue.

### Virulence and colonization assay

Virulence and colonization assays were performed as described previously (22). In brief, bacteria grown to an OD_600_ of 1–1.5 in GCB medium were harvested, washed, and resuspended in PBS to a final concentration of 10^9^ CFU/mL. Ten 3-day-old ducklings per group were injected with a 200-µL dose of 10^9^ CFU bacteria in the leg using a syringe. Survival was recorded for all strains for 7 days. For the colonization assay, 3-day-old ducklings (10/group) were injected with 5 × 10^8^ CFU of WT, *∆ruhB* and *∆ruhB^C^* in the leg. At 24 h post-infection, six selected ducklings were euthanized, and tissues were collected, weighed, ground, diluted, and plated on blood agar plates to determine the number of bacteria per milliliter or gram of tissue.

### Statistical analysis

Statistical analyses were performed using GraphPad Prism 9 software for Windows. Statistical significance was ascertained using Student’s *t*-test. *P*-values of <0.05 were considered significant.

### Bioinformatic analysis

RhuB and its homologous sequences were obtained from the NCBI database. Homologous proteins were selected based on an identity greater than 40% and coverage greater than 90%. Multiple sequence alignments were performed using the Cluster Omega tool (39). A phylogenetic tree was constructed by the neighbor-joining method (bootstrap replicates ×1,000) using the MEGA7 software (40), followed by subsequent refinement using iTOL v6 (https://itol.embl.de).

## Data Availability

The nucleotide sequence of RA CH-1 was deposited in GenBank under the accession number CP003787. Accession numbers for TonB-dependent receptors are listed in Table S3. GenBank accession numbers pertaining to RhuB homologs are listed in Table S4. The data sets generated and/or analyzed in this study are available from the corresponding authors upon reasonable request.

## SUPPLEMENTAL MATERIAL

Tables S1 and S2, Fig. S1 to S4 spectrum.03133-23-s0001.doc Supplemental material.

Table S3 spectrum.03133-23-s0002.xlsx The accession numbers of TonB-dependent receptors.

Table S4 spectrum.03133-23-s0003.xlsx The GenBank accession numbers of RhuB homologs.

ASM does not own the copyrights to Supplemental Material that may be linked to, or accessed through, an article. The authors have granted ASM a non-exclusive, world-wide license to publish the Supplemental Material files. Please contact the corresponding author directly for reuse.
