# Supplementary material for: Functional characterization of RhuB as a second TonB2-dependent hemin receptor in Riemerella anatipestifer CH-1
Source: Microbiol Spectr. 2024 Feb 20;12(4):e03133-23. doi: 10.1128/spectrum.03133-23 (PMC10986502; doi:10.1128/spectrum.03133-23)
Supplement: Tables S1 and S2, Fig. S1 to S4 — Supplemental material. [file spectrum.03133-23-s0001.doc]

**Supplementary Material**

**Table S1. Strains and plasmids used in this study.**

| ***E. coli* strains** | **Genotype** | | **Source or reference** |
| --- | --- | --- | --- |
| DH5α | | F^-^*,φ80dlacZ ΔM15,Δ*(*lacZYA-argF )U169, deoR, recA1, endA1* ,*hsdR17 (rK^-^, mK^+^), phoA, supE44, λ^-^, thi-1, gyrA96*, *relA1* | Laboratory collection |
| S17-1 | | *hsdR17 recA1* RP4-2-tet::Mu-1kan::Tn7; Sm^R^ | (1) |
| BL21(DE3) | | *E. coli* B F^-^ *dcm ompT hsdS(rB^-^ mB^-^) gal lon* λ(DE3 [*lacI lacUV5-T7* gene *1 ind1 sam7 nin5*]) | Laboratory collection |
| BL21(DE3) pET32a::*rhuB* | | BL21(DE3), pET32a carrying *rhuB* from RA CH-1, Amp^R^ | This study |
| BL21(DE3) pET32a::*fur* | | BL21(DE3), pET32a carrying *fur* from RA CH-1, Amp^R^ | This study |
| JP313 pBAD24::*tonB1* | | JP313, pBAD24::*tonB1*, Amp^R^ | (2) |
| ***Riemerella anatipestifer* strains** | | **Phenotype or genotype** | **Source or reference** |
| RA CH-1 | | *R. anatipestifer serotype* 1, Km^R^, Erm^R^ | Laboratory collection |
| RA CH-1 pLMF03 | | RA CH-1, pLMF03, Cfx^R^ | This study |
| RA CH-1*∆rhuB* pLMF03 | | RA CH-1, *rhuB* mutant, pLMF03, Cfx^R^ | This study |
| RA CH-1*∆rhuB* pLMF03::*rhuB* | | RA CH-1, *rhuB* mutant, pLMF03::*rhuB,* Cfx^R^ | This study |
| RA CH-1*∆fur* | | RA CH-1, *fur* mutant | (3) |
| RA CH-1*∆fur* pLMF03::*fur* | | RA CH-1, *fur* mutant, pLMF03::*fur*, Cfx^R^ | (3) |
| RA CH-1*∆tonB1* pLMF03 | | RA CH-1, *tonB1* mutant, pLMF03, Cfx^R^ | (4) |
| RA CH-1*∆tonB2* pLMF03 | | RA CH-1, *tonB2* mutant, pLMF03, Cfx^R^ | (4) |
| RA CH-1*∆tonB1∆rhuB* pLMF03 | | RA CH-1, *tonB1* mutant, *rhuB* mutant, pLMF03, Cfx^R^ | This study |
| RA CH-1*∆tonB1∆rhuB* pLMF03::*rhuB* | | RA CH-1, *tonB1* mutant, *rhuB* mutant, pLMF03::*rhuB*, Cfx^R^ | This study |
| RA CH-1*∆tonB2∆rhuB* pLMF03 | | RA CH-1, *tonB2* mutant, *rhuB* mutant, pLMF03, Cfx^R^ | This study |
| RA CH-1*∆tonB2∆rhuB* pLMF03::*rhuB* | | RA CH-1, *tonB2* mutant, *rhuB* mutant, pLMF03::*rhuB*, Cfx^R^ | This study |
| RA CH-1*∆rhuA* pLMF03 | | RA CH-1, *rhuA* mutant, pLMF03, Cfx^R^ | (4) |
| RA CH-1*∆rhuB∆rhuA* pLMF03 | | RA CH-1, *rhuB* mutant, *rhuA* mutant, pLMF03, Cfx^R^ | This study |
| RA CH-1*∆rhuB∆rhuA* pLMF03::*rhuA* | | RA CH-1, *rhuB* mutant, *rhuA* mutant, pLMF03::*rhuA*, Cfx^R^ | This study |
| RA CH-1*∆tonB1∆rhuR* pLMF03 | | RA CH-1, *tonB1* mutant, *rhuR* mutant, pLMF03, Cfx^R^ | (4) |
| RA CH-1*∆tonB2∆rhuR* pLMF03 | | RA CH-1, *tonB2* mutant, *rhuR* mutant, pLMF03, Cfx^R^ | (4) |
| RA CH-1*∆tonB1∆rhuR∆rhuB* pLMF03 | | RA CH-1, *tonB1* mutant, *rhuR* mutant, *rhuB* mutant, pLMF03, Cfx^R^ | This study |
| RA CH-1*∆tonB2∆rhuR∆rhuB* pLMF03 | | RA CH-1, *tonB2* mutant, *rhuR* mutant, *rhuB* mutant, pLMF03, Cfx^R^ | This study |
| RA CH-1*∆tonB1∆rhuR∆rhuB* pLMF03::*rhuB* | | RA CH-1, *tonB1* mutant, *rhuR* mutant, *rhuB* mutant, pLMF03::*rhuB*, Cfx^R^ | This study |
| RA CH-1*∆tonB2∆rhuR∆rhuB* pLMF03::*rhuB* | | RA CH-1, *tonB2* mutant, *rhuR* mutant, *rhuB* mutant, pLMF03::*rhuB*, Cfx^R^ | This study |
| **Plasmids** | | **Genotype** | **Source or reference** |
| pLMF03 | | Shuttle plasmid, *ermF* promoter, *ori*ColE1, *ori*pRA0726, Amp^R^, Cfx^R^ | (2) |
| pLMF03::*fur* | | pLMF03 carrying *fur* from RA CH-1, Amp^R^, Cfx^R^ | (3) |
| pET32a | | T7 promoter, His-tag, Amp^R^ | Laboratory collection |
| pET32a::*rhuB* | | pET32a carrying *rhuB* adding His tag from RA CH-1, Km^R^ | This study |
| pET32a::*fur* | | pET32a carrying *fur* adding His tag from RA CH-1, Km^R^ | This study |
| pLMF03::*rhuB* | | pLMF03 carrying *rhuB* from RA CH-1, Amp^R^, Cfx^R^ | This study |
| pLMF03::*rhuA* | | pLMF03 carrying *rhuA* from RA CH-1, Amp^R^, Cfx^R^ | (4) |
| pOES | | Suicide plasmid carrying *EXpheS**, Amp^R^, Cfx^R^ | (5) |
| pOES::*rhuB* up-down | | pOES carrying *rhuB* upstream and *rhuB* downstream from RA CH-1, Amp^R^, Cfx^R^ | This study |
| pOES::*rhuA* up-down | | pOES carrying *rhuA* upstream and downstream from RA CH-1, Amp^R^, Cfx^R^ | This study |

Km^R^, kanamycin resistance; Erm^R^, erythromycin resistance; Amp^R^, ampicillin resistance; Cfx^R^, cefoxitin resistance.

**Table S2.** Primers used in this study.

| **Primer** | **Organism** | **Sequence ( 5'－3')** |
| --- | --- | --- |
| RhuB upP1 | RA CH-1 | CCGCTCGAGCGGCCTCAACACCCAAGAATTAAGC |
| RhuB upP2 | RA CH-1 | CTTGGCATACCTGGTCTGTATCCTGGCGAATGAAGCTACTGAAAC |
| RhuB downP1 | RA CH-1 | GTTTCAGTAGCTTCATTCGCCAGGATACAGACCAGGTATGCCAAG |
| RhuB downP2 | RA CH-1 | GACTAGTCCTATCATTACTGAGTATCAGGATAAGG |
| Cfx P1 | pLMF03 | GGTGCTGCAATGTTGATG |
| Cfx P2 | pLMF03 | CCGCTAAGGTATAACTG |
| RhuB CompP1 | RA CH-1 | ACGCGTCGACGTCGGCCATAGCGGTTTAAACAAACTATCTCAACCTACTGCAAAG |
| RhuB CompP2 | RA CH-1 | CCGCTCGAGCGGTTATAAGCTGATTCTTACACCCACATTTAC |
| Fur ComP1 | RA CH-1 | CATGCCATGGAACATCAAGAGAAAG |
| Fur ComP2 | RA CH-1 | GGACTAGTCCTTATGCTTTTTTATGACCGTAG |
| RhuB ExpP1 | RA CH-1 | GGAATTCCATATGAAAAAACAACTTTTACCTC |
| RhuB ExpP2 | RA CH-1 | CGGGGTACCCCGTTAGTGGTGGTGGTGGTGGTGTAAGCTGATTCTTACACCCAC |
| RhuB qRTP1 | RA CH-1 | GGCTGATAACTCTAACGAAATGCTG |
| RhuB qRTP2 | RA CH-1 | CGTTGAACTGAAGCCCTTGTG |
| RecA qRTP1 | RA CH-1 | TGAAACTAGGTGATGGTACG |
| RecA qRTP2 | RA CH-1 | CTTAGGATAACCGCCTACTC |
| Fur ExpP1 | RA CH-1 | CGCCATATGGAATATCAAGGAAAAG |
| Fur ExpP2 | RA CH-1 | CCGGAATTCCGGTTAGTGGTGGTGGTGGTGGTGTGCCTTTTTATGACCGTAG |
| RhuA upP1 | RA CH-1 | CCGCTCGAGCGGGTCAGATTTTTATGAATCCTC |
| RhuA upP2 | RA CH-1 | CTTACTTCCGCTGGTCTGATAAGGTAGCTACCGTTAGAAATCC |
| RhuA downP1 | RA CH-1 | GGATTTCTAACGGTAGCTACCTTATCAGACCAGCGGAAGTAAG |
| RhuA downP2 | RA CH-1 | GACTAGTCAAAGAAATCTGCACCTGCCCAAG |
| 16S qRTP1 | RA CH-1 | ATGCGAAAGGAGGATTGC |
| 16S qRTP2 | RA CH-1 | TTACACCTCAAATACCTC |
| RhuBpromoterP1 | RA CH-1 | GGGGCTATCTCTATGAGATA |
| RhuBpromoterP2 | RA CH-1 | TATACTTTATTTAGAATTG |

**Fig S1. Expression of RhuB is regulated by iron and Fur levels.**

**
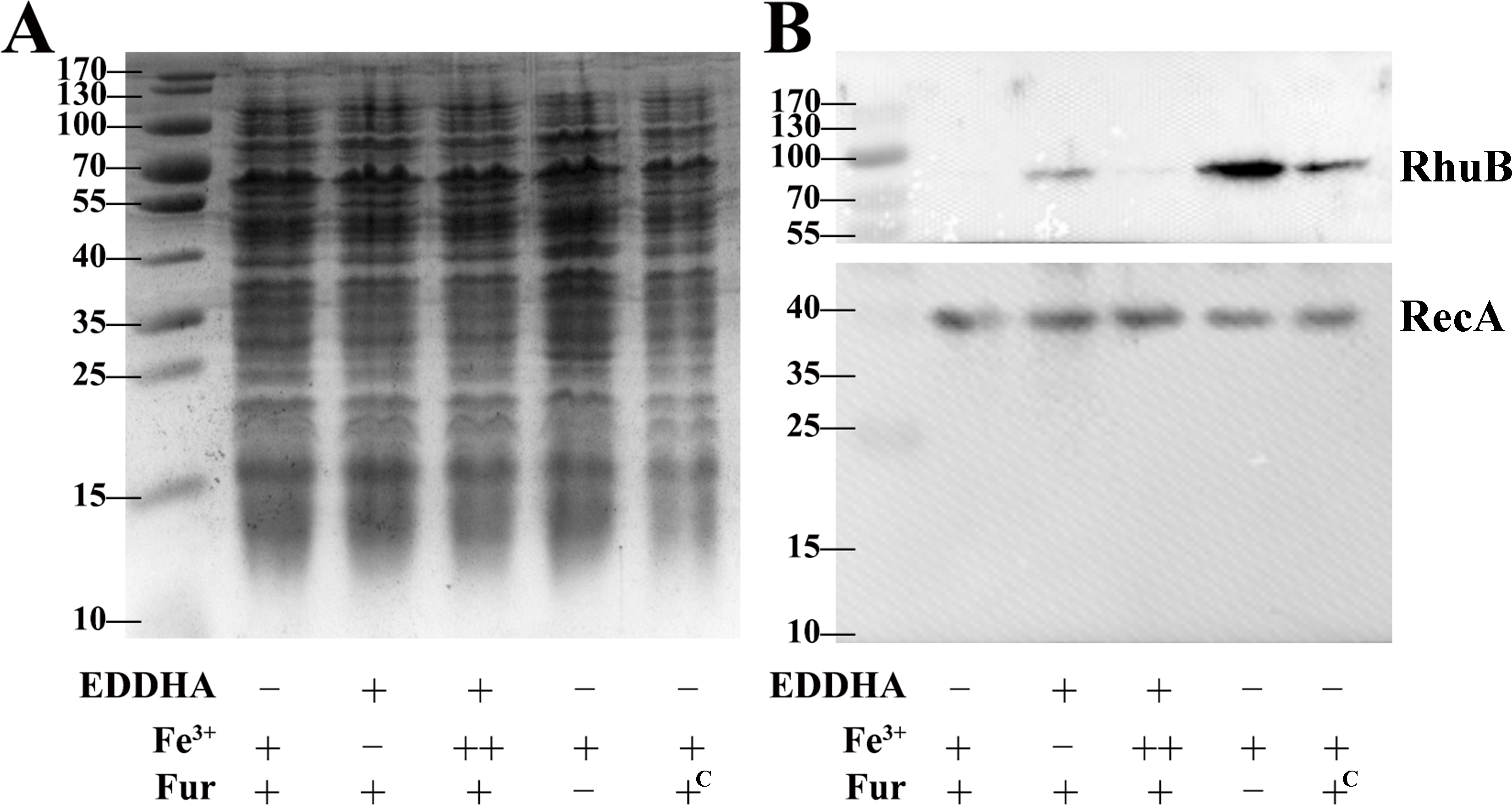
**

**Fig S1.** **Expression of RhuB is regulated by iron and Fur levels. (A)** RA CH-1 bacterial cells were cultured in the GCB medium (Lane 1), in GCB containing 120 μM EDDHA (Lane 2), in GCB containing 120 μM EDDHA supplemented with 100 μM Fe(NO_3_)_3_ (Lane 3). RA CH-1Δ*fur* pLMF03 and RA CH-1Δ*fur* pLMF03::*fur* bacterial cells were cultured in the GCB medium (Lanes 4 and 5). The samples were subjected to SDS-PAGE, and one gel was stained with Coomassie brilliant blue. **(B)** The remaining gel was transferred onto a PVDF membrane and subjected to immunoblotting using a specific anti-RhuB antibody. RecA was used as the internal reference.

**Fig S2. Subcellular localization of RhuB in RA CH-1**

**
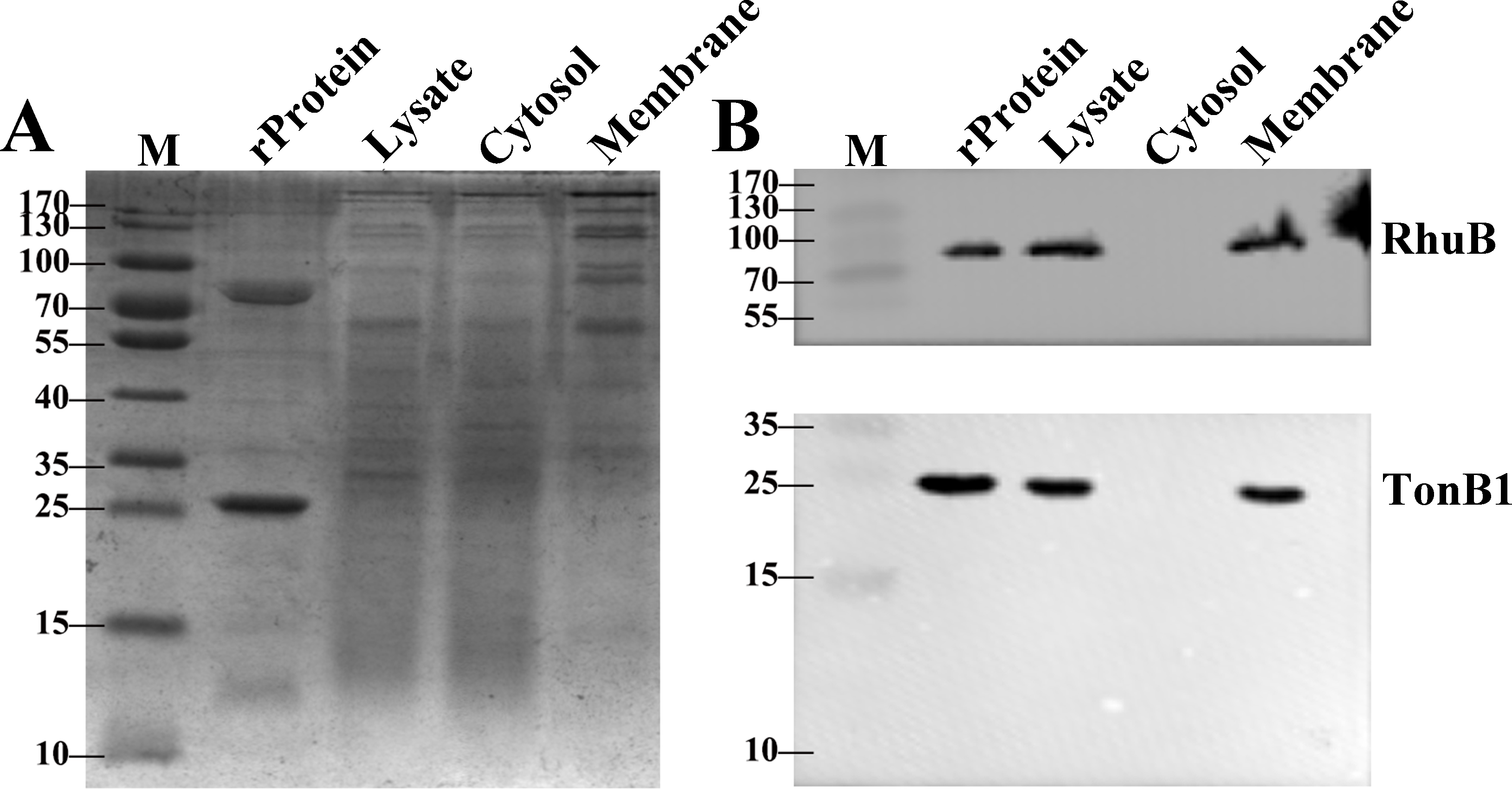
**

**Fig S2.** **Subcellular localization of RhuB in RA CH-1*.*** RhuB was detected using polyclonal antibodies in the RA CH-1 lysate, cytosol, and membrane. Cellular fractions were loaded onto SDS-PAGE gels. **(A)** After electrophoresis, the gel was stained with Coomassie brilliant blue. **(B)** Proteins from the other gel were transferred to a PVDF membrane and subjected to immunoblot analysis using a specific anti-RhuB antibody. The subcellular location of the membrane protein TonB1 was used as a positive control. **rProtein:** Purified recombinant protein used for comparison. **Lysate:** whole cell lysate of RA CH-1. **Cytosol:** Cytoplasmic protein fraction of RA CH-1. **Membrane:** membrane protein fraction of RA CH-1.

**Fig S3.** **Growth curves of Δ*tonB1*, Δ*tonB2* and its derivative strains under different conditions.**

**
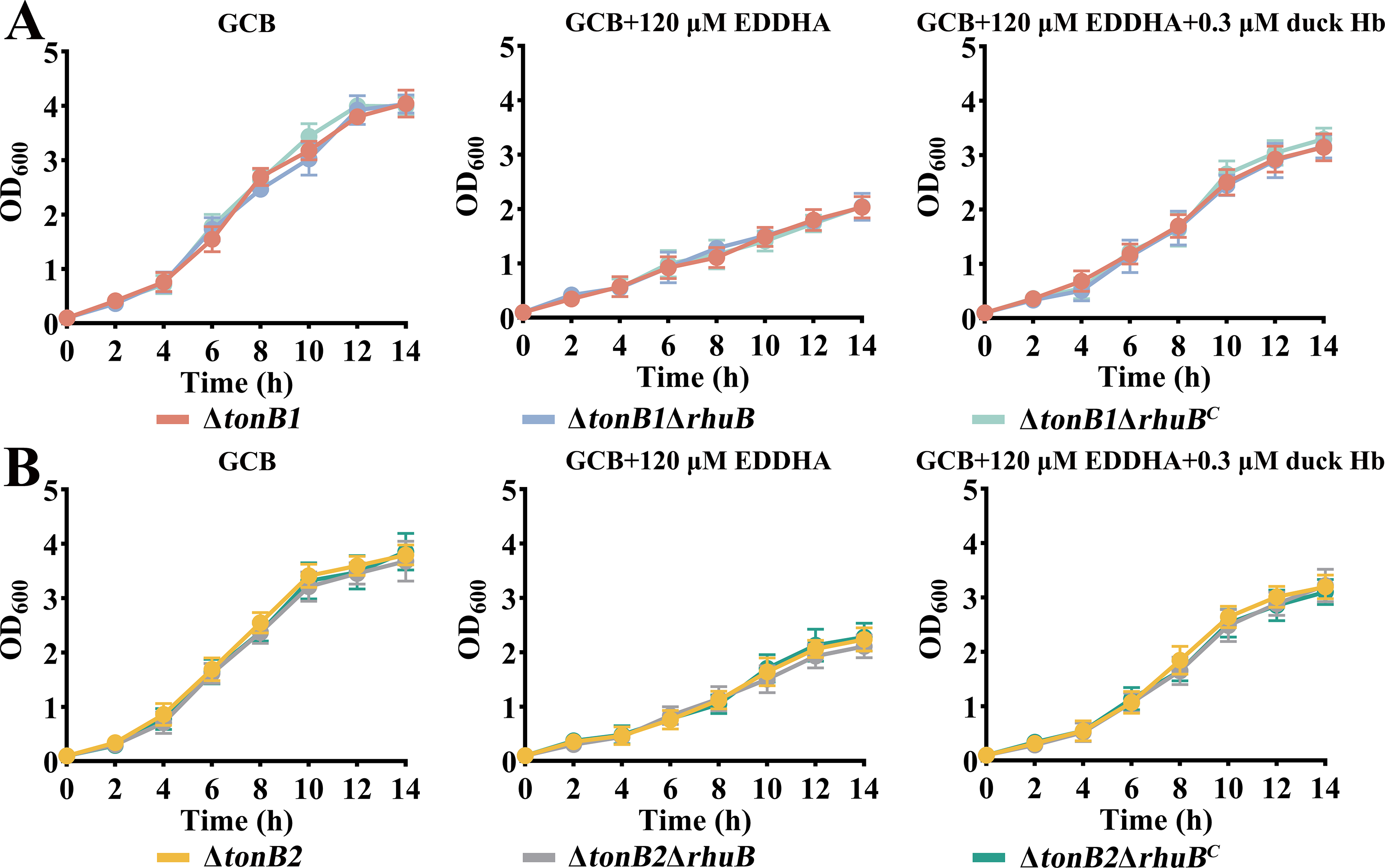
**

**Fig S3. Growth curves of** **Δ*tonB1*, Δ*tonB2* and its derivative strains under different conditions.** **(A)** The growth curves of RA CH-1Δ*tonB1* pLMF03 (Δ*tonB1*), RA CH-1Δ*tonB1*Δ*rhuB* pLMF03 (Δ*tonB1*Δ*rhuB*), RA CH-1Δ*tonB1*Δ*rhuB* pLMF03::*rhuB* (Δ*tonB1*Δ*rhuB^C^*) in the GCB liquid medium, GCB combined with 120 μM EDDHA, and GCB combined with 120 μM EDDHA and supplemented with 0.3 μM duck hemoglobin. **(B)** The growth curves of RA CH-1Δ*tonB2* pLMF03 (Δ*tonB2*), RA CH-1Δ*tonB2*Δ*rhuB* pLMF03 (Δ*tonB2*Δ*rhuB*), RA CH-1Δ*tonB2*Δ*rhuB* pLMF03::*rhuB* (Δ*tonB2*Δ*rhuB^C^*) in the GCB liquid medium, GCB combined with 120 μM EDDHA, and GCB combined with 120 μM EDDHA and supplemented with 0.3 μM duck hemoglobin. The data shown are the averages and standard deviations derived from three experiments.

**Fig S4. The multiple sequence alignment of RhuB and its homologous proteins.**

**
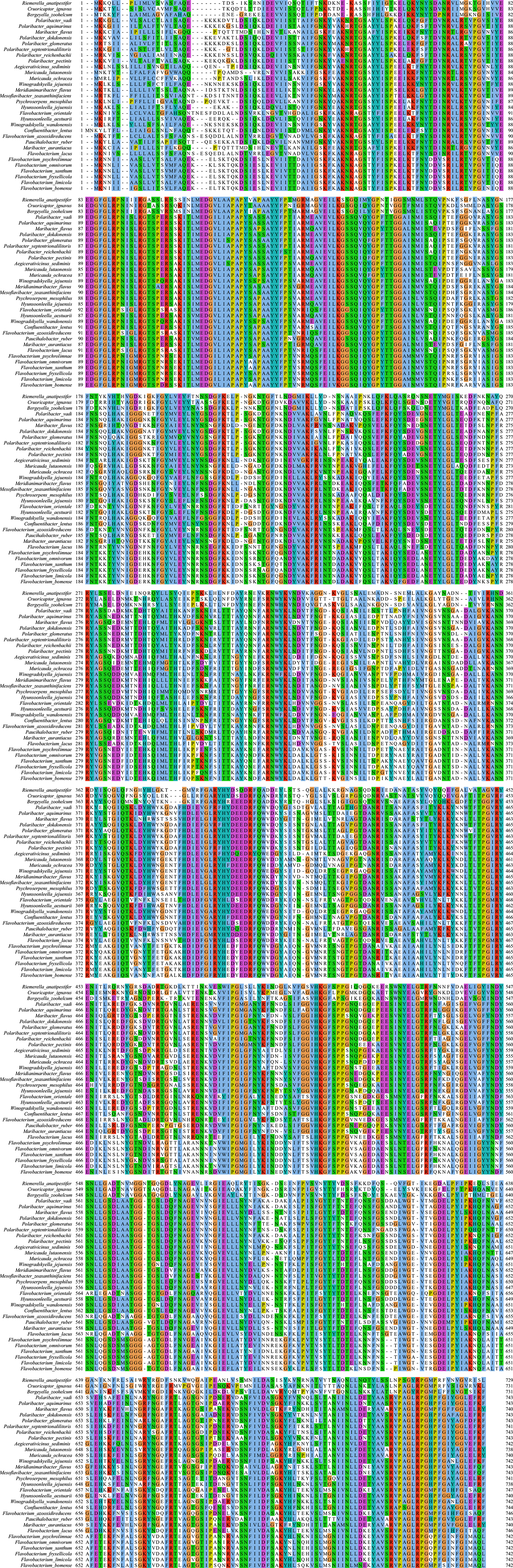
**

**Fig S4. The multiple sequence alignment of RhuB and its homologous proteins.** The sequences were obtained from NCBI, aligned using ClustalW, and the image was edited using Jalview.

**References**

1. Simon R, Priefer U, Puhler A. 1983. A broad host mobilization system for in vivo genetic engineering: Transposon mutagenesis in Gram-negative bacteria. Bio/Technolgy 1:37-45.

2. Liu M, Wang M, Zhu D, Wang M, Jia R, Chen S, Sun K, Yang Q, Wu Y, Chen X, Biville F, Cheng A. 2016. Investigation of TbfA in Riemerella anatipestifer using plasmid-based methods for gene over-expression and knockdown. Sci Rep 6:37159.

3. Huang M, Liu M, Liu J, Zhu D, Tang Q, Jia R, Chen S, Zhao X, Yang Q, Wu Y, Zhang S, Huang J, Ou X, Mao S, Gao Q, Sun D, Wang M, Cheng A. 2021. Functional characterization of Fur in iron metabolism, oxidative stress resistance and virulence of Riemerella anatipestifer. Vet Res 52:48.

4. Liu M, Liu S, Huang M, Wang Y, Wang M, Tian X, Li L, Yang Z, Wang M, Zhu D, Jia R, Chen S, Zhao X, Yang Q, Wu Y, Zhang S, Huang J, Ou X, Mao S, Gao Q, Sun D, Yu YL, Cheng A. 2021. An Exposed Outer Membrane Hemin-Binding Protein Facilitates Hemin Transport by a TonB-Dependent Receptor in Riemerella anatipestifer. Appl Environ Microbiol 87:e0036721.

5. Liu M, Huang Y, Liu J, Biville F, Zhu D, Wang M, Jia R, Chen S, Zhao X, Yang Q, Wu Y, Zhang S, Chen X, Liu Y, Zhang L, You Y, Yu Y, Cheng A. 2018. Multiple genetic tools for editing the genome of Riemerella anatipestifer using a counterselectable marker. Appl Microbiol Biotechnol 102:7475-7488.
